# Herbal adaptogens combined with protein fractions from bovine colostrum and hen egg yolk reduce liver TNF-α expression and protein carbonylation in Western diet feeding in rats

**DOI:** 10.1186/1743-7075-11-19

**Published:** 2014-04-23

**Authors:** C Brooks Mobley, Ryan G Toedebusch, Christopher M Lockwood, Alexander J Heese, Conan Zhu, Anna E Krieger, Clayton L Cruthirds, John C Hofheins, Joseph M Company, Charles E Wiedmeyer, Dae Y Kim, Frank W Booth, Michael D Roberts

**Affiliations:** 1 School of Kinesiology, Auburn University, Auburn, AL, USA; 2 Department of Biomedical Sciences, University of Missouri, Columbia, MO, USA; 3 4LIFE Research, Sandy, UT, USA; 4 Department of Veterinary Pathobiology, University of Missouri, Columbia, MO, USA; 5 Department of Nutrition and Exercise Physiology, University of Missouri, Columbia, MO, USA; 6 Department of Medical Pharmacology and Physiology, University of Missouri, Columbia, MO, USA; 7 Dalton Cardiovascular Research Center, University of Missouri, Columbia, MO, USA

**Keywords:** Liver, Inflammation, Oxidative stress, RNA-seq

## Abstract

**Background:**

We examined if a purported anti-inflammatory supplement (AF) abrogated Western-diet (WD)-induced liver pathology in rats. AF contained: 1) protein concentrates from bovine colostrum and avian egg yolk; 2) herbal adaptogens and antioxidants; and 3) acetyl-L-carnitine.

**Methods:**

Nine month-old male Brown Norway rats were allowed *ad libitum* access to WD for 41–43 days and randomly assigned to WD + AF feeding twice daily for the last 31–33 days (n = 8), or WD and water-placebo feeding twice daily for the last 31–33 days (n = 8). Rats fed a low-fat/low-sucrose diet (CTL, n = 6) for 41–43 days and administered a water-placebo twice daily for the last 31–33 days were also studied. Twenty-four hours following the last gavage-feed, liver samples were analyzed for: a) select mRNAs (via RT-PCR) as well as genome-wide mRNA expression patterns (via RNA-seq); b) lipid deposition; and, c) protein carbonyl and total antioxidant capacity (TAC). Serum was also examined for TAC, 8-isoprostane and clinical chemistry markers.

**Results:**

WD + AF rats experienced a reduction in liver Tnf-α mRNA (-2.8-fold, p < 0.01). Serum and liver TAC was lower in WD + AF versus WD and CTL rats (p < 0.05), likely due to exogenous antioxidant ingredients provided through AF as evidenced by a tendency for mitochondrial SOD2 mRNA to increase in WD + AF versus CTL rats (p = 0.07). Liver fat deposition nor liver protein carbonyl content differed between WD + AF versus WD rats, although liver protein carbonyls tended to be lower in WD + AF versus CTL rats (p = 0.08). RNA-seq revealed that 19 liver mRNAs differed between WD + AF versus WD when both groups were compared with CTL rats (+/- 1.5-fold, p < 0.01). Bioinformatics suggest that AF prevented WD-induced alterations in select genes related to the transport and metabolism of carbohydrates in favor of select genes related to lipid transport and metabolism. Finally, serum clinical safety markers and liver pathology (via lesion counting) suggests that chronic consumption of AF was well tolerated.

**Conclusions:**

AF supplementation elicits select metabolic, anti-inflammatory, and anti-oxidant properties which was in spite of WD feeding and persisted up to 24 hours after receiving a final dose.

## Background

Non-alcoholic fatty liver disease, also referred to as nonalcoholic steatohepatitis (NASH), is a condition whereby there is an increased deposition of fat in the liver resulting in inflammation and liver damage [1,2]. NASH has accompanied the escalating prevalence in obesity with nearly 37% of the U.S. population reporting to have this disease [3]. Causes of NASH include but are not limited to poor dietary choices [4-6] and the lack of adequate daily physical activity [7-9]. With regard to dietary causes of NASH, rodent models have clearly demonstrated that diets high in sugar and fat, which is typical of ‘Westernized’ diets (WD), are mechanistically linked to the development of this condition [10-12].

Given that a critical feature of NASH includes an increase in liver oxidative stress, antioxidant supplementation may be a viable strategy in slowing the progression of this disease. Indeed, sparse evidence suggests that antioxidant supplementation may be beneficial in the treatment and progression of diet-induced fatty liver disease [13]. Likewise, antioxidants are known to protect cellular structures against damage from free radicals and reactive oxygen species of lipid peroxidation [14-16]; both of which are known to be elevated during NASH [17]. Inflammation is another critical feature of NASH, and pro-inflammatory cytokines have been found to be highly expressed due to lipid peroxidation via oxidative stress [15,18,19]; an effect which can lead to downstream inflammation and liver damage [20]. Hence, along with reducing oxidative stress, a dietary strategy to reduce liver inflammation that occurs during diet-induced NASH is also warranted in order to reduce disease progression.

The purpose of the current study was to determine if WD-induced increases in markers of NASH development were reduced in adult Brown Norway rats after 30 days of dietary supplementation with a purported anti-inflammatory and antioxidant (AF) supplement. Specific AF ingredients included the following: 1) bovine colostrum and hen egg yolk extract; 2) herbal adaptogens and antioxidants; and 3) acetyl-L-carnitine. We hypothesized that AF supplementation would reduce WD-induced markers of NASH development in light of the plethora of data suggesting that these ingredients possess antioxidant and anti-inflammatory properties [16,21-29]. In order to holistically examine the molecular effects that AF supplementation exhibited on liver physiology in the presence of WD feeding, RNA deep-sequencing (RNA-seq) and advanced bioinformatics on liver samples were also performed. Likewise, clinical chemistry serum markers were assessed in order to examine clinical safety as well as other potential metabolic effects AF supplementation exhibited in lieu of WD *ad libitum* feeding.

## Methods

### Animals and dietary feeding paradigm

All experimental protocols were approved by the University of Missouri’s Animal Care and Use Committee. Sixteen 9-month-old male Brown Norway rats (Charles River Laboratory, O’ Fallon, Missouri) were assigned to one of the following two groups: 1) *ad libitum* WD + AF feeding twice daily (n = 8); or 2) *ad libitum* WD + water-vehicle placebo feeding twice daily (n = 8). In addition, six rats were fed a 14% protein, low-fat/low sugar diet (CTL) and were administered a water-vehicle placebo over the feeding period in order to examine if the feeding protocol elicited metabolic stress upon the liver. Of note, rats were singly housed in standard rat cages in temperature-controlled animal quarters (21°C) with a 0700–1900 light: 1900–0700 dark cycle that was maintained throughout the experimental period.

Rats were provided 41–43 days of *ad libitum* WD or CTL feeding in total. During days 1–10, rats were provided their respective diets only as a run-in period. During the remaining 31–33 days afterward, AF rats were gavage-fed one human equivalent dose (human dose: 1,410 mg/dose, human eq. rat dose: 52 mg; from methods of Reagan-Shaw et al. [30]) of the supplement (4Life Transfer Factor Renuvo™, 4Life Research, Sandy, UT, USA) dissolved in 1 ml of tap water under light isoflurane anesthesia, twice per day. WD and CTL rats were gavage-fed 1 ml of tap water under light isoflurane anesthesia, twice per day. The active ingredients present within the purported AF supplement included a proprietary blend of: 1) bovine colostrum and hen egg yolk extract; 2) herbal adaptogens and antioxidants [*Rhaponticum carthamoides* (root) extract, *Schisandra chinensis* (fruit) extract, *Withania somnifera* (root) extract, *Camellia sinensis* (leaf) extract, *Curcuma longa* (rhizome) extract, piperine, resveratrol]; and 3) Acetyl-L-carnitine. As AF is a proprietary blend, we chose not to disclose explicit ingredient doses. However, the manufacturer provided our laboratory with a third-party laboratory certificate of analysis, which confirmed the identity, composition, potency and purity of the formulation. Specifically, product identity, heavy metals and physical testing were performed by Bio Medical Research (Sandy, UT), pesticide testing was performed by Flora Research Laboratories (Grants Pass, OR), and microbiological testing was performed by Advanced Laboratories (Salt Lake City, UT).

WD (TD.88137; Harlan Laboratories) contained the following macronutrient profile: 4.5 kcal/g, 15.2% protein (expressed as %kcal), 42.7% carbohydrate (expressed as %kcal), 42.0% fat (expressed as %kcal). Of note, this diet has been used in prior rodent studies to elicit NASH [31,32]. CTL (LabDiet_®_ Certified CR 14% protein rodent diet) contained the following macronutrient profile: 3.5 kcal/g, 16.1% protein (expressed as %kcal), 69.3% carbohydrate (expressed as %kcal), 14.6% fat (expressed as %kcal). Additionally, WD contained a high amount of sucrose (34% by weight), whereas CTL contained a low amount of sucrose (1.2% by weight). Food intakes were carefully measured weekly (i.e., monitoring cage bottoms for morsels, etc.) and total caloric intakes were calculated for the duration of the study.

### Sacrificial procedures

Rats were administered either AF or a water placebo up to 24 hours prior to sacrifice. On the day of sacrifice, animal cages were removed from the animal quarters between 0800–0900 and food was removed from each cage. Sacrifices took place between 1400–1900 and rats were sacrificed under CO_2_ gas in their home cages in order to minimize stress. Whole blood was subsequently removed via heart sticks using a 21-gauge needle and syringe, placed in a serum separator tube, centrifuged at 3200 rev/min for 5 min, and serum was aliquoted into multiple 1.7 ml microcentrifuge tubes for subsequent biochemical assays. Four median lobe liver sections (~50–100 mg) were subsequently removed using standard dissection techniques and were preserved in the following manners for each of these respective assays: 1) frozen in Trizol for real-time polymerase chain reaction (PCR) and RNA-seq analyses; 2) flash frozen in liquid nitrogen for Oil Red-O staining; 3) refrigerated in 10% phosphate buffered formalin for hematoxylin and eosin (H&E) staining; 4) flash-frozen in liquid nitrogen for protein carbonyl and total antioxidant capacity (TAC) assessment.

### Serum and liver biochemical assays

Serum samples were assayed for TAC and 8-isoprostane via spectrophotometric methods (Cayman Chemical, Ann Arbor, MI) according to manufacturer instructions, and general clinical chemistry markers as previously described [33].

Approximately 50 mg of frozen liver samples were homogenized in 50 mM phosphate buffer with 1 mM EDTA. Total protein content was determined spectrophotometrically using a BCA kit (Thermo Scientific, Waltham, MA), and samples were assayed for TAC and protein carbonyls according to manufacturer instructions (Cayman Chemical, Ann Arbor, MI). Of note, respective TAC and protein carbonyl values were corrected for total protein loaded for each assay.

### Liver real-time PCR methods

Liver samples preserved in Trizol were lysed using a high-speed shaking apparatus (Tissuelyser LT; Qiagen, Germantown, MD) with RNase-free stainless steel beads. RNA was subsequently separated into phases using the Trizol method according to manufacturer’s instructions and isolated/DNase treated with columns (Macherey-Nagel, Bethlehem, PA, USA) and 1 μg of RNA was reverse transcribed into cDNA for real time PCR analyses. Real-time PCR was performed using SYBR-green-based methods with gene-specific primers [superoxide dismutase (SOD)1, SOD2, p47phox, p67phox, interleukin (Il)-6, and tumor necrosis factor (Tnf)-α] designed using primer designer software (Primer Premier, Premier Biosoft, Palo Alto, CA). The forward and reverse primer sequences are as follows: SOD1 FP (5′ → 3′): TGTGTCCATTGAAGATCGTGTGA, SOD1 RP (5′ → 3′): TCTTGTTTCTCGTGGACCACC, SOD2 FP (5′ → 3′): TTAACGCGCAGATCATGCA, SOD2 RP (5′ → 3′): CCTCGGTGACGTTCAGATTGT, p47phox FP (5′ → 3′): ACGCTCACCGAGTACTTCAACA, p47phox RP (5′ → 3′): TCATCGGGCCGCACTTT, p67phox FP (5′ → 3′): GCTTCGGAACATGGTGTCTAAGA, p67phox RP (5′ → 3′): AGAGTCAGGCAGTAGTTTTTCACTTG, Il-6 FP (5′ → 3′): AGGGAGATCTTGGAAATGAGAAAA, Il-6 RP (5′ → 3′): TCATCGCTGTTCATACAATCAGAA, Tnf-α FP (5′ → 3′): CCCAGAAAAGCAAGCAACCA, Tnf-α RP (5′ → 3′): CCTCGGGCCAGTGTATGAGA.

### Liver histology

Liver Oil Red-O staining was performed for lipid counting using standard histology techniques on frozen tissue. H&E staining was performed for lesion counting using standard histology techniques on formalin-fixed tissue. Following each respective histological procedure, digital images were acquired via light microscopy using an Olympus BX60 photomicroscope at × 10 magnification (Olympus, Melville, NY). Lipid droplet accumulation was then quantified using an automated split color channel function within ImageJ (NIH, Bethesda, MD), and H&E liver lesions were counted and scored by a certified pathologist (Dr. Dae Kim, College of Veterinary Medicine, University of Missouri).

### Liver RNA-seq methods

#### RNA isolation and cDNA preparation

Two micrograms of liver RNA were sent to the University of Missouri’s DNA Core for RNA-seq procedures. Briefly, high RNA integrity of each sample was confirmed using the BioAnalyzer 2100 automated electrophoresis system (Bio-Rad, Hercules, CA, USA) prior to cDNA library construction. cDNA library preparation was subsequently performed using the manufacturer’s protocol with reagents supplied in Illumina’s TruSeq RNA sample preparation kit v2. Briefly, poly-A containing mRNA was purified from 2 μg of total RNA, RNA was fragmented, double-stranded cDNA was generated from fragmented RNA and the index containing sample identifier adapters were ligated to the ends. The final construct of each purified library was evaluated using the BioAnalyzer 2100 automated electrophoresis system, quantified with the Qubit fluorometer using the quant-iT HS dsDNA reagent kit (Invitrogen, Life Technologies, Grand Island, NY), and diluted according to Illumina’s standard sequencing protocol for sequencing on the HiSeq 2000.

#### Illumina sequencing of NAc cDNA and statistical analyses of RNA-seq data

RNA-seq procedures occurred at the University of Missouri DNA Core and are described in more detail elsewhere [34]. Briefly, following cDNA library construction, samples were loaded onto a flow cell where clusters of each oligo were replicated. Following this procedure, flow cells were placed in the sequencer and fluorescently-labeled bases were attached to the complementary bases of each sequence. The Illumina Genome Analyzer recorded 50 bp reads. Reads were trimmed to ensure adaptor sequence removal and tiled to a custom reference using NextGENe v1.92 (SoftGenetics, State College, PA).

### Statistics and bioinformatics for liver RNA-seq data

Differential gene expression patterns were analyzed for annotated genes between the WD + AF, WD, and CTL groups using reads per kilobase per million mapped reads (RPKM) values. Differentially expressed liver mRNAs were considered to be significant when WD + AF/CTL fold-change values > ±1.5 and an independent *t*-test p-value < 0.01, whereas the same gene was not differentially expressed between WD/CTL (p > 0.05). Of note, these analyses thresholds are similar to other recent RNA-seq publications, which have used a 1.5-to-2.0 fold-change cut-off and similar p-value significant thresholds [35-37]. Genes that were found to be altered with AF supplementation were subsequently entered into Ingenuity Pathway Analysis (Qiagen) to elucidate gene networks and/or biological functions within the liver that were altered with AF supplementation.

### Statistics for non-RNA-seq data

Unless otherwise stated, dependent variables are presented as mean ± standard error and between-group comparisons for dependent variables were performed using one-way ANOVAs. Significance was set at an alpha level of 0.05. When significance was observed, Tukey post-hoc comparisons were performed in order to determine specific between-group differences.

## Results

### Between-treatment phenotypes following the feeding intervention

**Figure 1:**
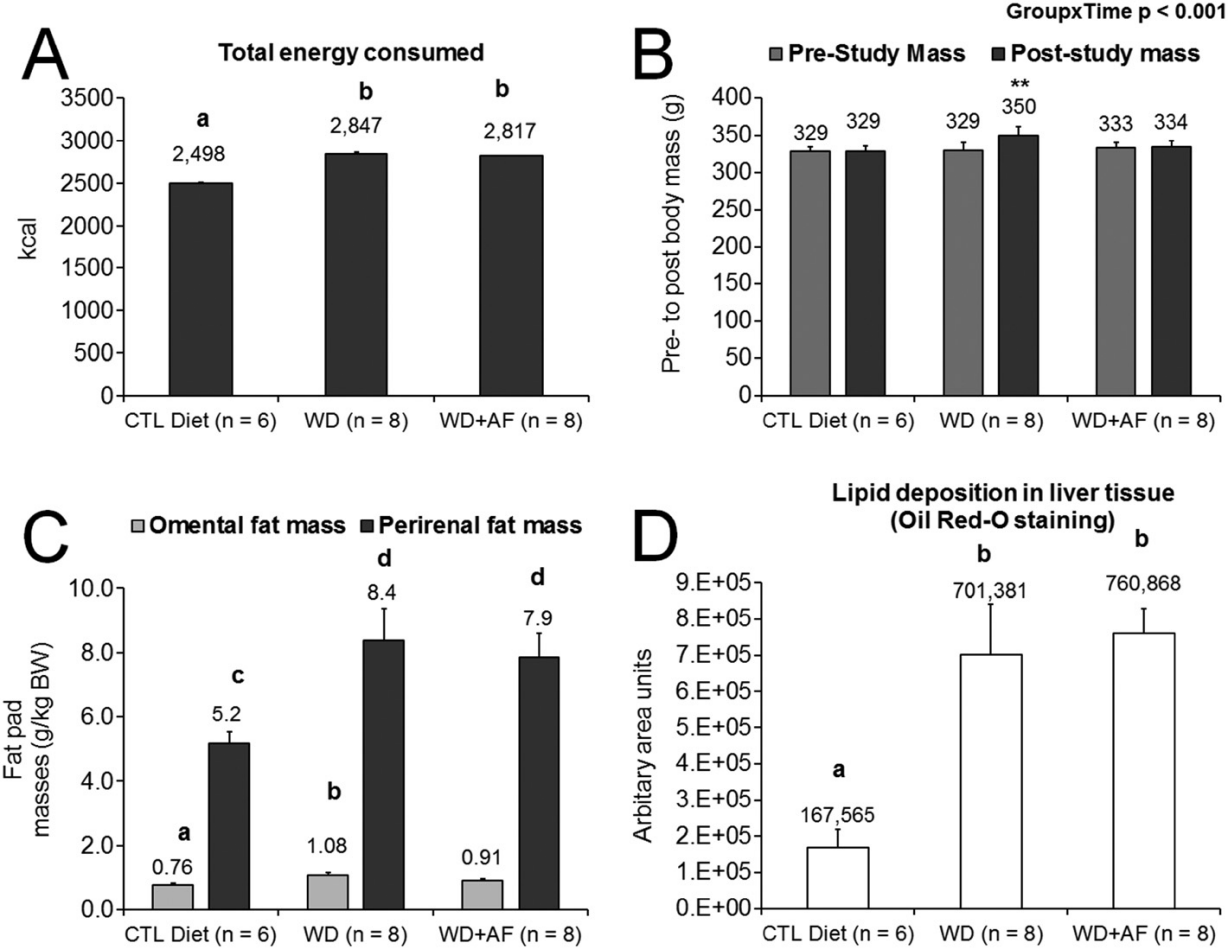
**Between-treatment phenotypes during and following the feeding intervention.** WD and WD + AF similarly increased Caloric intake compared to CTL rats **(A)**. WD rats experienced an increase in body mass pre- to post- intervention **(B)**, although omental fat pad masses, perirenal fat pad masses and liver fat deposition assessed during necropsies were not different between treatments **(C/D)**. Abbreviations and symbols: CTL = control low-fat/low-sugar 14% diet, WD = Western diet, WD + AF = Western diet + anti-inflammatory supplement; different superscript letters represents differences between groups (p < 0.05); In **A**, different superscript letters represents differences between groups (b > a, p < 0.05); In **B**, **represents a significant pre- to post-change in body mass within groups (p < 0.01); In **C**, different superscript letters represents differences between groups (for omental fat mass b > a, p < 0.05; for perirenal fat mass d > c); In **D**, different superscript letters represents differences between groups (b > a, p < 0.05).

During the feeding intervention, WD + AF and WD consumed significantly more kilocalories than CTL (p < 0.05; Figure 1A). Interestingly, pre- to post-intervention body masses remained unaltered within the CTL and WD + AF rats, whereas WD rats weighed more after the intervention compared to their baseline body mass values (p < 0.01, Figure 1B). However, post-intervention omental and perirenal fat pad masses were similar between WD + AF and WD rats (Figure 1C) suggesting that the prevention of body mass gain with AF supplementation may have been due to alterations in hydration, losses in other fat pad masses (i.e., subcutaneous), and/or losses in other tissue mass (i.e., liver, visceral organ, muscle mass, and/or other tissue masses). Hence, this finding warrants further interrogation.

Contrary to our hypotheses, WD + AF rats did not result in a decreased deposition of liver lipid accumulation (Figure 1D). Specifically, the only lesions that occurred amongst all treatments were signs of lipidosis, and the degree of lipidosis included the following (*data not graphed*): 1) non-existent in CTL rats; 2) mild in WD + AF rats (n = 8); and 3) minimal (n = 1), mild (n = 6), and moderate (n = 2) in WD rats. While trichromatic staining is the preferred method for liver fibrosis detection, other literature has demonstrated that significant fibrosis can be detected with H&E staining [38]. Nonetheless, our pathologist did not detect appreciable liver fibrosis formation within or between groups.

**Figure 2:**
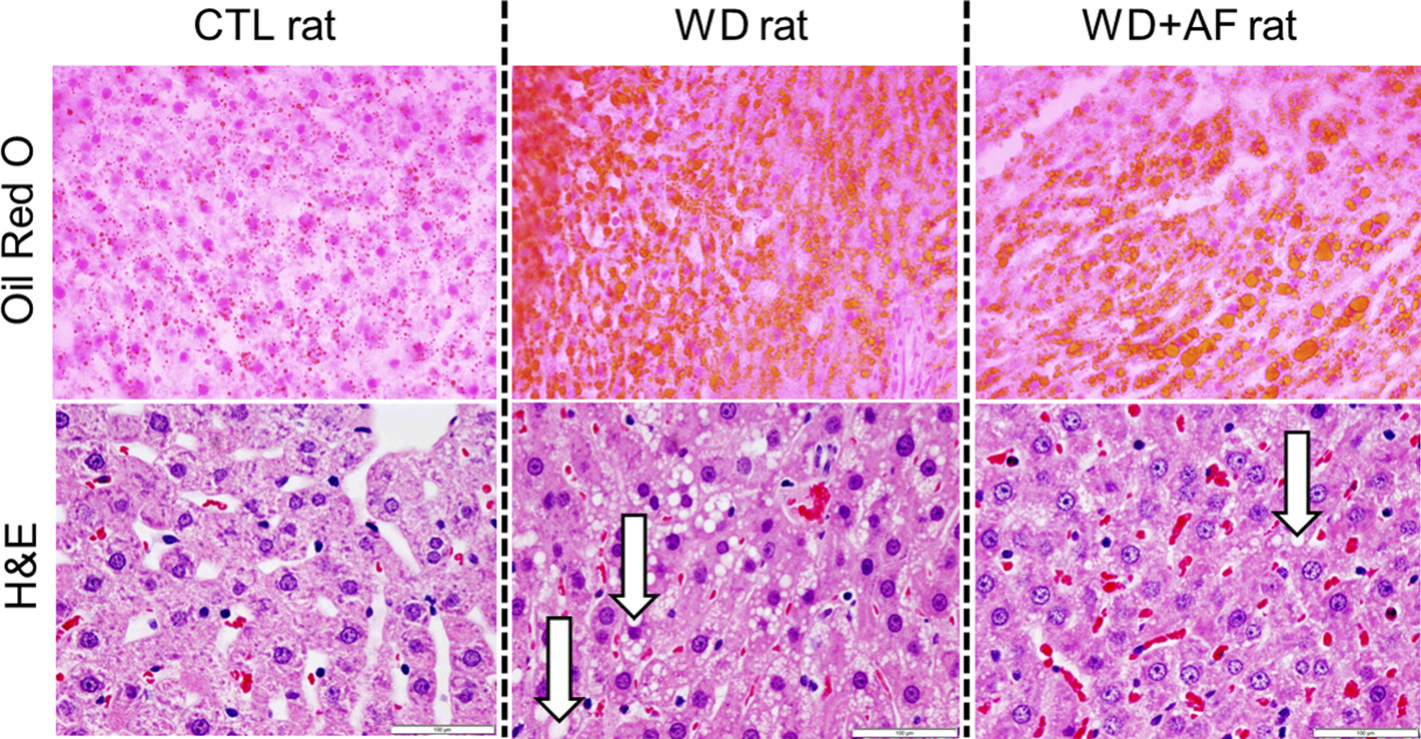
**Representative Oil Red O and H&E photomicrographs.** Images were captured using the 10× objective. Upper photomicrographs are Oil Red O images whereby red-orange globules are lipid droplets. Lower photomicrographs are H&E images whereby cytosolic lipid vacuoles are depicted by white arrows in the WD and WD + AF rats; note that all CTL rats presented minimal signs of these vacoules. White bars on H&E stains represent 100 μm markers.

Representative images of Oil Red O and H&E liver stains are presented in Figure 2.

### Markers of antioxidant capacity and liver oxidative stress following the feeding intervention

**Figure 3:**
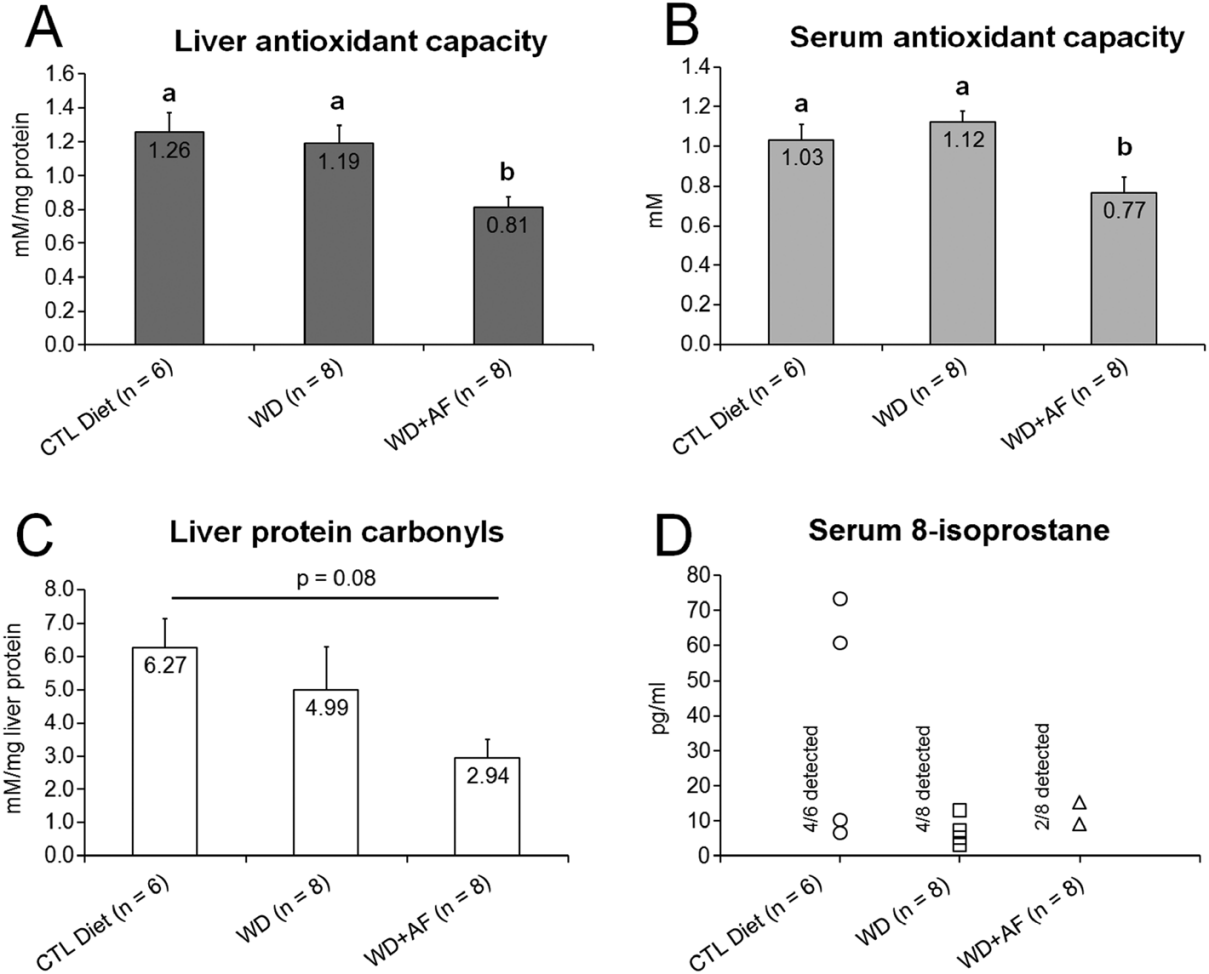
**Markers of antioxidant capacity and oxidative stress following the feeding intervention.** WD + AF rats experienced a decrease in liver **(A)** and serum total antioxidant capacity **(B)**, although there was a paradoxical tendency for liver protein carbonyls to decrease in WD + AF rats **(C)**. Only 25% of WD + AF rats presented detectable levels of serum 8-isoprostane, a marker of systemic oxidative stress, whereas 50 and 66% of the WD and CTL animals presented detectable levels of this marker **(D)**. Abbreviations and symbols: CTL = control low-fat/low-sugar 14% diet, WD = Western diet, WD + AF = Western diet + anti-inflammatory supplement; In **A/B**, different superscript letters represents differences between groups (a > b, p < 0.05).

Following the feeding intervention, WD + AF rats presented lower levels of liver (Figure 3A) and serum TAC (Figure 3B). However, there was a paradoxical tendency for liver protein carbonyls to decrease in WD + AF rats compared to CTL rats (p = 0.08, Figure 3C). Interestingly, only 25% of WD + AF rats presented detectable serum 8-isoprostane levels, whereas 50% of WD and 66% of CTL rats presented detectable levels of this marker (*no statistics run*; Figure 3D).

### Liver real-time PCR results

**Figure 4:**
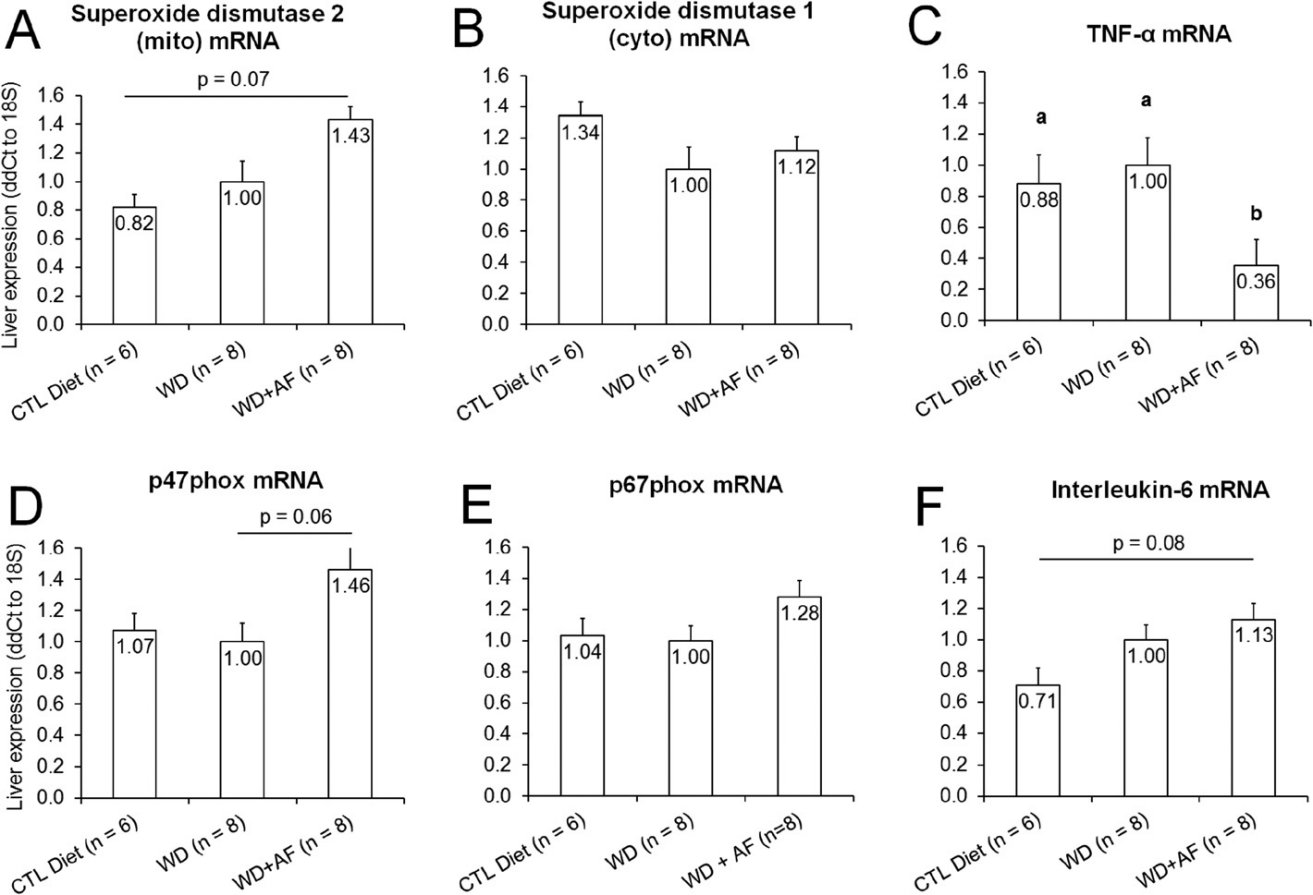
**Effects of CTL, WD, and WD + AF feeding on SOD2 mRNA (A), SOD2 mRNA (B), Tnf-α mRNA (C), p47phox mRNA (D), p67phox mRNA (E), and IL-6 mRNA (F).** WD + AF rats experienced a decrease in liver Tnf-α mRNA expression **(C)** compared to WD and CTL rats; WD + AF rats tended to increase liver SOD2 mRNA **(A)** and Il-6 mRNA compared to CTL **(F)** rats; WD + AF rats tended to increase liver p47phox mRNA compared to WD **(C)** rats. Abbreviations and symbols: CTL = control low-fat/low-sugar 14% diet, WD = Western diet, WD + AF = Western diet + anti-inflammatory supplement; In C, different superscript letters represents differences between groups (a > b, p < 0.05).

Following the feeding intervention, WD + AF rats tended to show an increase SOD2 mRNA (Figure 4A). WD + AF rats also tended to show an increase in liver p47phox mRNA compared to WD rats (p = 0.06, Figure 4E), as well as a tendency for Il-6 to increase compared to CTL rats (p = 0.08, Figure 4F). Interestingly, WD + AF rats experienced a decrease in liver Tnf-α mRNA compared to WD and CTL (Figure 4C) rats.

### Liver RNA-seq and bioinformatics suggests that AF may shift liver away from carbohydrate metabolism

RNA-seq revealed that, out of 11,634 mRNAs that possessed RPKM values greater than 2.0, seven transcripts were up-regulated (fold-change +1.5, p < 0.01) and 12 transcripts were down-regulated (fold-change -1.5, p < 0.01) in WD + AF versus WD rats when both groups were compared to CTL rats (Table 1).

**Table 1:** Liver RNA-seq results from WD + AF rats versus WD rats when both groups were compared to CTL rats (p < 0.01)

**Gene**	Gene function*	**WD + AF/CTL Fold-change**	**WD/CTL Fold-change**
Efna5	Functions in pancreatic islet cells to regulate glucose-stimulated insulin secretion	**-2.20**	-1.37
Ahr	Mediates biochemical and toxic effects of halogenated aromatic hydrocarbons	**-1.93**	-1.35
Pck1	Main control point for the regulation of gluconeogenesis	**-1.90**	-1.31
Sall1	Transcriptional repressor involved in organogenesis	**-1.88**	-1.41
Rasgef1b	Guanine nucleotide exchange factor with specificity for Rap2a and other Ras family proteins	**-1.87**	-1.35
Acadsb	Member of the acyl-CoA dehydrogenase family of enzymes that catalyze the dehydrogenation of acyl-CoA derivatives in the metabolism of fatty acids or branch chained amino acids.	**-1.65**	-1.31
Tmem56	Unknown function	**-1.62**	-1.09
Ivns1abp	Protects cells from cell death induced by actin destabilization	**-1.58**	-1.08
Slc2a2	Mediates facilitated bidirectional glucose transport (a.k.a. Glut2)	**-1.56**	-1.22
Hspa13	Members of this protein family play a role in the processing of cytosolic and secretory proteins, as well as in the removal of denatured or incorrectly-folded proteins	**-1.56**	-1.11
Bach1	Plays important roles in coordinating transcription activation and repression by Mafk	**-1.52**	-1.28
C21orf91	Unknown function	**-1.51**	-1.16
Adcy9	Membrane bound enzyme that catalyses the formation of cyclic AMP from ATP	**1.55**	1.32
Cd36	Binds long chain fatty acids and may function in the transport and/or as a regulator of fatty acid transport	**1.61**	1.55
Slc39a4	Plays an important role in cellular zinc homeostasis as a zinc transporter	**1.69**	1.33
Foxa2	Encodes a member of the forkhead class of DNA-binding proteins, and can control the expression of liver-specific genes such as albumin	**1.69**	1.29
Ces2	Participate in fatty acyl and cholesterol ester metabolism	**1.73**	1.41
Ltb	Plays a specific role in immune response regulation	**1.83**	1.49
Nr0b2	Inhibits transcriptional activity of Neurod1 on E-box-containing promoter	**2.99**	2.39

Legend: bold-faced fold-change values indicate that genes that were altered by WD + AF but were not altered with WD when both conditions compared to CTL rats. * = descriptions adapted from http://www.genecards.org.

**Figure 5:**
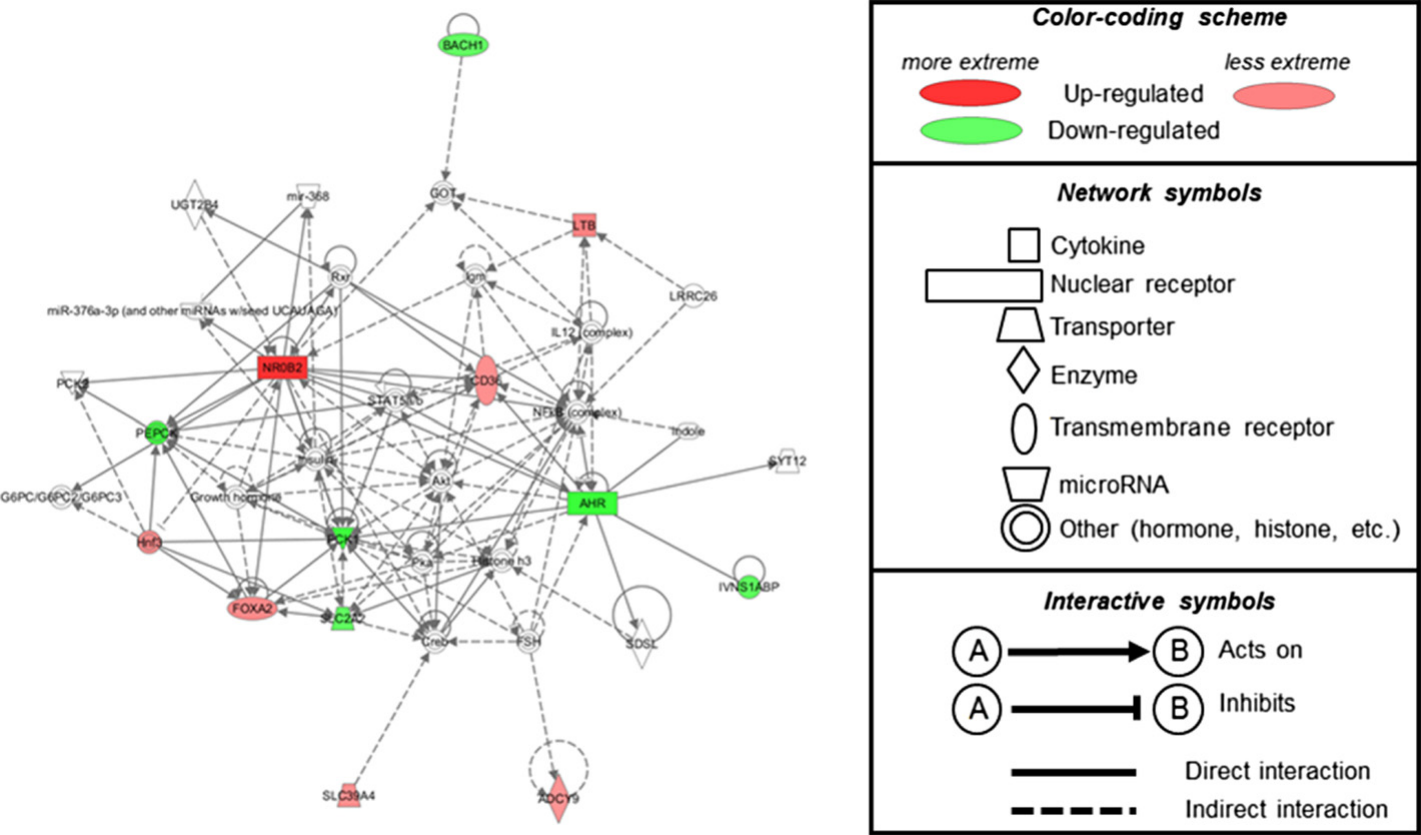
**Top IPA liver gene network altered in WD + AF versus WD rats compared to CTL rats.** Notably, relative to WD/CTL comparisons, WD + AF/CTL comparisons demonstrated a significant up-regulation in Cd36 (fatty acid transport) and down-regulation of Pck1 and Slc2a2 (both of which are involved with gluconeogenesis and glucose transport, respectively). Left inset is a diagram of the top liver gene network significantly altered (up- and down-regulated): ‘*Gene Expression, Carbohydrate Metabolism, Molecular Transport*’ (score: 29; up-regulated mRNAs: Ltb, Nr0b2, Cd36, Foxoa2, Hnf3; down-regulated mRNAs: Pepck, Pck1, Slc2a2, Ahr, Ivns1abp). Right inset is the legend for transcript function, degree of up-regulation/down-regulation, and interactive symbols.

Bioinformatics suggested that the top biological function activated in WD + AF versus WD rats included: ‘*Increased Concentration of Lipid*’ (Ahr, Cd36, Efna5, Foxa2, Nr0b2, Pck1, Slc2a2 were up- or down-regulated with WD + AF/CTL and did not change with WD/CTL; categorical function p-value ranges = 4.11E-8 to 2.32E-9). The top associated liver gene network altered by WD + AF but were not altered with WD when both conditions were compared to CTL included: ‘*Gene Expression, Carbohydrate Metabolism, Molecular Transport*’ (score: 29; up-regulated mRNAs: Ltb, Nr0b2, Cd36, Foxoa2, Hnf3; down-regulated mRNAs: Pepck, Pck1, Slc2a2, Ahr, Ivns1abp) (Figure 5).

### Serum clinical safety markers between groups following the feeding intervention

Serum clinical safety markers following the intervention are presented in Table 2. Serum glucose was significantly lower in CTL compared to WD and WD + AF rats (p < 0.05). Serum alkaline phosphatase was significantly lower in WD and WD + AF versus CTL rats (p < 0.05). Serum calcium was significantly lower in WD + AF and CTL rats when compared to WD rats (p < 0.05). Serum creatinine was significantly lower in WD + AF and WD rats when compared to CTL rats (p < 0.05). There were no statisitical differences in other clinical safety markers between groups.

**Table 2:** Serum clinical safety markers between groups following the feeding intervention

**Variable**	**CTL (n = 6)**	**WD (n = 8)**	**WD + AF (n = 8)**	**ANOVA p-value**
*Metabolic markers*				
Glucose (mg/dl)	143 ± 11^a^	205 ± 13^b^	204 ± 13^b^	**0.005**
Cholesterol (mg/dl)	74.7 ± 2.7	80.8 ± 2.0	77.8 ± 2.2	0.21
Triglycerides (mg/dl)	73.3 ± 4.9	123.1 ± 21.0	131.4 ± 18.5	0.088
*Markers of liver health and function*				
Total Protein (g/dl)	7.2 ± 0.1	7.1 ± 0.1	7.2 ± 0.2	0.81
Albumin (g/dl)	3.47 ± 0.03	3.36 ± 0.03	3.39 ± 0.04	0.18
Total bilirubin (mg/dl)	0.10 ± 0.00	0.10 ± 0.00	0.10 ± 0.00	1.00
Alkaline Phosphatase (U/L)	133 ± 12^a^	93 ± 9^b^	89 ± 7^b^	**0.006**
Alanine aminotransferase (U/L)	60.5 ± 11.3	75.3 ± 15.6	60.9 ± 7.6	0.62
Gamma-glutamyltransferase (U/L)	< 3	< 3	< 3	N/A
Globulin (g/dl)	3.70 ± 0.03	3.74 ± 0.03	3.78 ± 0.08	0.67
*Other serum markers*				
Sodium (mEq/L)	150 ± 3	147 ± 1	147 ± 1	0.29
Potassium (mEq/L)	5.57 ± 0.21	5.60 ± 0.27	5.09 ± 0.12	0.17
Chloride (mEq/L)	98.6 ± 1.5	99.6 ± 0.5	99.5 ± 0.4	0.19
Total CO_2_ (mEq/L)	42.8 ± 1.0	40.9 ± 0.8	40.5 ± 0.8	0.18
Calcium (g/dl)	11.1 ± 0.1^a^	11.4 ± 0.1^b^	11.2 ± 0.1^a,b^	**0.043**
Phosphorus (mg/dl)	6.12 ± 0.12	6.63 ± 0.19	6.21 ± 0.06	**0.038**
Blood urea nitrogen (mg/dl)	16.0 ± 0.6	14.4 ± 0.5	14.3 ± 0.6	0.082
Creatinine (mg/dl)	0.49 ± 0.01^a^	0.45 ± 0.01^a,b^	0.44 ± 0.01^b^	**0.016**
Creatine Kinase (U/L)	64.3 ± 5.4	69.8 ± 8.2	79.4 ± 7.2	0.37

Notes: Tukey post-hoc comparisons performed when ANOVA p < 0.05; different letter superscripts indicate significant differences between groups.

"Legend: Bold-faced values are significant between-treatment differences when Tukey post-hoc comparisons performed (p < 0.05); different letter superscripts indicate significant differences between groups."

## Discussion

Here we demonstrate that, in spite of of WD-induced fatty liver development, AF supplementation: 1) reduces Tnf-α mRNA; 2) tended to reduce liver protein carbonyl levels and did decrease 8-isoprostane levels when these animals were compared to CTL rats, albeit the latter marker was also reduced in WD rats as well; 3) tended to increase liver SOD2 mRNA, which encodes the mitochondrial superoxide dismutase antioxidant, when these animals were compared to CTL rats; and 4) prevented WD-induced alterations in select genes related to the transport and metabolism of carbohydrates in favor of select genes related to lipid transport and metabolism. However, AF supplementation did not affect fatty liver development in the presence of WD feeding. Finally, serum clinical chemistry markers and liver histopathology demonstrated that sub-chronic (30 days), twice daily AF supplementation was well-tolerated, and that the significant between-group effects were observed up to 24 hours after receiving a final treatment dose. More in depth discussion of these findings are presented herein.

WD + AF rats experienced a significant reduction in liver Tnf-α mRNA compared to WD and CTL rats. Chicanine, a major lignan present within *Schisandra chinensis*, an active ingredient in AF, was recently reported by Chen et al. [39] to significantly down-regulate LPS-induced expression of Tnf-α mRNA, as well as other pro-inflammatory cytokines in murine macrophages. Similarly, Dushkin et al. [40] recently showed that eight weeks of daily oral *Rhaponticum carthamoides* extract, an active present in AF, significantly lowered serum Tnf-α levels in high-fat diet fed six-month old male Wistar rats. Green tea extract, another active ingredient in AF, has been shown to protect against NASH in ob/ob mice by decreasing the expression of adipose tissue lipogenic genes, improving hepatic antioxidant defenses and decreasing liver Tnf-α [21,22]. Likewise, Li et al. [23] demonstrated that supplementing a high fat diet with resveratrol, an active ingredient in AF, amerliorated fibrosis, insulin resistance, glucose tolerance, dysregulated lipid metabolism, oxidative stress, and pro-inflammatory responses. Rivera et al. [24] similarly revealed that elevated liver biomarkers of NASH were significanlty lower in obese Zucker rats following 8-week treatment of resveratrol. The authors further explained that resveratrol was able to increase adiponectin protein content and lower Tnf-α protein content in the visceral adipose tissue of rats. Therefore, a decrease in liver Tnf-α mRNA may have been due to schisandra, rhaponticum, resveratrol, green tea extract, and/or other ingredients present in AF.

Serum and liver TAC were significantly lower in WD + AF versus WD and CTL rats. We posit that the latter findings could be a result of the exogenous antioxidant ingredients being provided through AF which, in turn, decreases the expression of total endogenous antioxidant capacity across multiple tissues. In this regard, Valtuena et al. [41] reported that diets high in total antioxidant capacity significantly improved markers of inflammation, but did not appreciably affect antioxidant status in healthy adults. Foods high in antioxidants have also been shown to prevent carotenoid secretion into circulation up to 24 hours following feeding [42]. Likewise, while a controversial topic [43], the possibility exists that exogenously provided dietary antioxidants hamper endogenous antioxidant systems [44]. However, the decrease in serum 8-isoprostane levels in the WD + AF rats versus CTL rats does provide a line of evidence suggesting that systemic oxidative stress was still lower in the former group while serum TAC was also reduced.

Liver protein carbonyl concentration within the mitochondria and nuclear compartments, but not within the soluble cellular compartments such as the cytosol have been linked to mammalian aging [44]; specifically, short-lived animals were shown to possess higher concentrations of liver protein carbonyls within the insoluble cellular components such as the mitochondria, and long-living animals were shown to possess reduced protein carbonyls within these cellular compartments. Interestingly, within the present investigation, total liver protein carbonyls tended to be lower in WD + AF versus CTL rats, which is suggestive of AF possessing potentially anti-oxidant properties in the liver. Though AF signficantly reduced serum and liver TAC, our study findings would seem to suggest that AF possesses antioxidant properties. This is of no surprise given that AF contains green tea extract and resveratrol. Chung et al. [22] reported that green tea extract inhibits ROS/RNS-mediated damage while reducing serum alanine aminotransferase (ALT) and decreasing lipid peroxidation and protein nitration. Rubiolo et al. [25] similarly revealed that resveratrol protects isolated primary rat hepatocytes from necrosis induced by oxidative stress. Gómez-Zorita et al. [27] demonstrated that a 6-week treatment of resveratrol significantly decreased lipid peroxidation, suggesting a protective antioxidant effect against obesity and steatosis-induced oxidative stress. It is noteworthy to mention, however, that WD feeding in the current study did not alter markers of oxidative stress when comparing WD versus CTL rats. This finding was likely due to the relatively sub-chronic WD feeding schedule. However, NASH development appears to have been occurring, as is evident from the observed liver fat deposition, though no overt signs of oxidative stress became apparent. Therefore, this finding suggests that, AF supplementation reduces liver protein carbonyl formation and may reduce systemic oxidative stress as evidenced by only 25% of the animals presenting detectable levels of serum 8-isoprostane compared to 50–66% of the rats within the other groups presenting detectable levels of this marker.

While liver TNF-α mRNA and oxidative stress markers were favorably altered with AF supplementation, liver lipid deposition remained unaltered. Pan et al. [45] reported similar findings when high-fat fed mice were provided either a water- or ethanol-extracted schisandra fruit extract for 13 days. Specifically, whereas hepatic steatosis was ameliorated and liver injury reduced by the schisandra treatment, significant increases in high and low density lipoproteins occurred without any observable changes occuring within measures of total cholesterol and triglycerides. Jwa et al. [28], however, demonstrated that supplementing a high fat diet with piperine, another active ingredient in AF, markedly decreased hepatic lipid desposition in rodents. Nagao et al. [29] similarly reported that dietary resveratrol has an anti-obesity effect in obese rats. While it is difficult to reconcile our findings with the aforementioned two investigations, this discrepancy may be due to dosaging differences and/or the relatively short length of the current study.

A unique aspect of this study was using RNA-seq as an unbiased approach of examining liver transcriptome-wide differences in WD + AF versus WD animals when both groups were compared to CTL rats. Bioinformatic analyses suggest that AF potentially prevented WD-induced alterations in select genes that lead to the transport and metabolism of carbohydrates in favor of lipid transport and metabolism. Interestingly, phosphoenolpyruvate carboxykinase (Pck1) was down-regulated with AF supplementation. Gomez-Valades et al. [46] have demonstrated that RNA interference against the Pck1 gene improves glycemic control, insulinemia and blood lipid levels in db/db mice. Therefore, the AF-induced downregulation of Pck1 may potentially lead to a longer-term stability in liver carbohydrate and fatty acid metabolism in the midst of WD feeding. Glut2 (Slc2a2) was also down-regulated with AF supplementation. Okamato et al. [47] have illustrated *in vitro* that hepatocytes presenting steatosis show an enhanced expression of Glut2 mRNA; this being an effect which the authors suggest may be associated with liver gluconeogenesis and insulin resistance. Therefore, while the WD-fed rats supplemented with or without AF presented high fasting glucose levels suggestive of impaired glucose tolerance, the AF-induced decrease in liver Glut2 mRNA may again be potentially beneficial with regard to longer-term effects on liver metabolism. This is in agreement with Dushkin et al. [40] that reported an improvement in glucose sensitivity and increased liver PPAR-α DNA-binding activity in male rats treated with rhaponticum extract for eight weeks. Cd36 was up-regulated with AF supplementation and functions in the uptake of long chain fatty acids along with oxidizing low-density lipoproteins. A plethora of evidence suggests that a lowered Cd36 expression is metabolically protective while overexpression is likely to result in metabolic complications [47,48]. However, evidence also exists suggesting that Cd36 may be integral in glucose metabolism and insulin control [49-51].

## Conclusions

In summary, these data suggest that AF elicits select metabolic, anti-inflammatory, and anti-oxidant properties which were in spite of WD feeding. While others have used similar animal numbers per experimental treatment with transcriptome-wide interrogations [52] and to study the effects of quercetin on liver physiology in obese rats [53], a limitation of this study was that we similarly used a relatively small number of animals per experimental condition. Other potential limitations to this study are that it was a sub-chronic feeding period compared to other prolonged feeding protocols (i.e., 20+ weeks), and post-intervention tissue samples were collected approximately 24 hours subsequent the final treatment dosing. With regard to the former point, we were able to observe markers indicative of the development of fatty liver without advanced pathology in WD and/or WD + AF rats (i.e., fibrotic lesion development). Finally, we did not delve into specific mechanisms as to how AF supplementation reduces liver TNF-α expression and protein carbonyl formation. Therefore, a more mechanistic interrogation and studying the longer-term effects of AF supplementation on liver physiology during WD feeding are warranted. More specifically, because the current findings as well as the aforementioned literature suggests that several ingredients within AF may be efficacious in reducing liver fat deposition and metabolic maladies that accompany a more prolonged WD feeding schedule.

## Competing interests

Besides CML, none of the authors have non-finacial and/or financial competing interests with/against 4Life Research, Inc. CML is employed by 4Life Research, but was primary responsible for AF formulation and he intellectually contributed to study design, RNA-seq analyses and data write-up. Therefore, all co-authors agreed that his intellectual input into this project warranted co-authorship.

## Authors’ contributions

RGT, CML, AJH, CZ, AEK, CLC, JCH, JMC, CEW, DYK, FWB, MDR: have made substantial contributions to conception and design, or acquisition of data, or analysis and interpretation of data. CBM, MDR: primarily were involved in drafting the manuscript or revising it critically for important intellectual content. CBM, RGT, CML, AJH, CZ, AEK, CLC, JCH, JMC, CEW, DYK, FWB, MDR: gave final approval of the version to be published. CBM, RGT, CML, AJH, CZ, AEK, CLC, JCH, JMC, CEW, DYK, FWB, MDR: agrees to be accountable for all aspects of the work in ensuring that questions related to the accuracy or integrity of any part of the work are appropriately investigated and resolved. All authors read and approved the final manuscript.

## Authors’ information

Department of Biomedical Sciences, University of Missouri, Columbia, MO, USA:

Location where MDR conducted the study as principal investigator (PI); data analysis done at PI’s current address.
